# The antiviral immune forces awaken in the cancer wars

**DOI:** 10.1371/journal.ppat.1008814

**Published:** 2020-09-03

**Authors:** Dohun Pyeon, Lexi Vu, Nicholas S. Giacobbi, Joseph A. Westrich

**Affiliations:** 1 Department of Microbiology and Molecular Genetics, Michigan State University, East Lansing, Michigan, United States of America; 2 Department of Microbiology, Immunology, and Pathology, Colorado State University, Fort Collins, Colorado, United States of America; University of Wisconsin Madison, UNITED STATES

## Introduction

Viruses are the most abundant microorganisms on earth, and it is estimated that there are at least 10 times more viruses than bacteria in the human microbiome [1]. Unlike bacteria, many viruses can permanently settle in a host cell, are minimally influenced by environmental changes, and can even be inherited when infecting germline cells. Many viruses have coevolved with host species for millions of years and have developed mechanisms to evade immune recognition and maintain an equilibrium state with the host immune system. Recent studies have shown that breaking this immune equilibrium can activate the host antiviral immune responses [2–6].

Current cancer immunotherapies targeting immune checkpoint molecules have limited efficacy in treatment of noninflamed tumors (so-called “cold” tumors) that show few infiltrating T cells. The absence of T-cell infiltration is largely caused by the lack of tumor antigens, antigen presentation, and/or abundance of immunosuppressive cells in the tumor microenvironment (TME) [7]. To convert immune “cold” tumors to inflamed “hot” tumors, a variety of different strategies are currently under investigation, including bispecific antibodies, chimeric antigen receptor (CAR) T cells, oncolytic viruses, and cyclic GMP-AMP synthase-stimulator of interferon genes (cGAS-STING) agonists [8, 9]. The high mutation loads in tumors often result in expression of mutation-associated neoantigens, which are recognized as foreign antigens to the host T cells. These neoantigens have been found to be associated with improved responses to immune checkpoint inhibitors (ICIs) [10]. Interestingly, when suppressed viral gene expression in a tumor is reactivated from the virome, they are similarly recognized as tumor neoantigens by host immune systems [6, 11]. These findings suggest that activating immune responses against viruses in the human virome might be an effective tool for cancer immunotherapies.

## Which viruses are included in the human virome for potential tumor neoantigens?

The human virome consists of a multitude of viruses, including eukaryotic viruses that infect human cells, viruses that infect prokaryotes (including bacteria and archaea), and viral genetic elements that reside within the host genome [12]. Although a significant portion of the human virome consists of bacteriophages, our current understanding of the virome mainly centers around eukaryotic viruses [13].

Human endogenous retroviruses (HERVs) are the most abundant viral elements in the human virome, making up over 8% of the human genome [14]. Previous studies have shown potential associations between increased HERV protein expression and human diseases [15, 16]. All HERVs exist in the human virome as permanently integrated elements in the host genome, which can be inherited by future generations through germline cells.

Similar to HERVs, human herpesvirus 6 (HHV-6) is integrated into the host germline chromosome and inherited in about 1% of human populations [17]. Several other types of herpesviruses such as cytomegalovirus (CMV) and Epstein-Barr virus (EBV) establish life-long persistence in the vast majority of humans without showing any clinical symptoms. However, if the immune system is compromised, in for example, immunodeficient patients, they are at a higher risk for developing severe diseases, such as cancers, from these viruses [18]. In addition, small DNA viruses, such as human papillomaviruses and polyomaviruses, have coevolved with host species and are also frequently found in the human virome [19]. Although a small subset of these viruses encodes potent viral oncoproteins, the majority of persistent infections of these viruses are found in asymptomatic hosts. Unlike retroviruses, DNA viruses such as herpesviruses, papillomaviruses, and polyomaviruses normally maintain their genomes as multicopy episomes in persistently infected cells. These viral episomes, maintained in a covalently closed circular form, are assembled into chromatin with histones for autonomous replication [20]. However, these viruses also occasionally integrate into the host genome, despite no benefits for their replication from viral integration.

For these viruses to establish life-long persistence in the human virome, it is essential to avoid recognition and elimination by host antiviral immunity. Accordingly, almost all viruses in the human virome evade immune recognition by limiting viral replication and gene expression. The lack of viral gene expression and/or host immune tolerance leads to immune silence or an immune equilibrium state in the host. Thus, viral antigens from reactivated viral gene expression in tumor cells could be recognized as tumor neoantigens to induce antitumor immune responses.

## How can endogenous retroviruses be reactivated to induce antitumor immunity?

Viral gene expression from HERVs are predominantly silenced by DNA methylation and histone modification, resulting in a transcriptionally inactive heterochromatic structure [21]. Thus, reactivating HERVs using epigenetic drugs has been proposed to trigger antitumor immune responses [22, 23].

### DNA methyltransferase inhibitors

Reversing DNA methylation with DNA methyltransferase inhibitors (DNMTi), such as decitabine, reactivates expression of HERV RNA transcripts. Detection of these transcripts by the host double stranded RNA sensors, such as the toll-like receptors (TLRs) and melanoma differentiation-associated protein 5 (MDA5), drives an interferon (IFN) response [2, 24]. Due to chromosomal residence in the germline, HERV proteins should be generally considered as “self” peptides with their cognate T cells deleted in the thymus during T-cell development. However, due to the effective epigenetic silencing of HERVs, HERV-specific T cells are not removed during T-cell development and can be activated by transcriptional induction of HERVs [23]. Consequently, reactivated HERVs in tumor cells can be recognized by host as tumor neoantigens.

**Fig 1:**
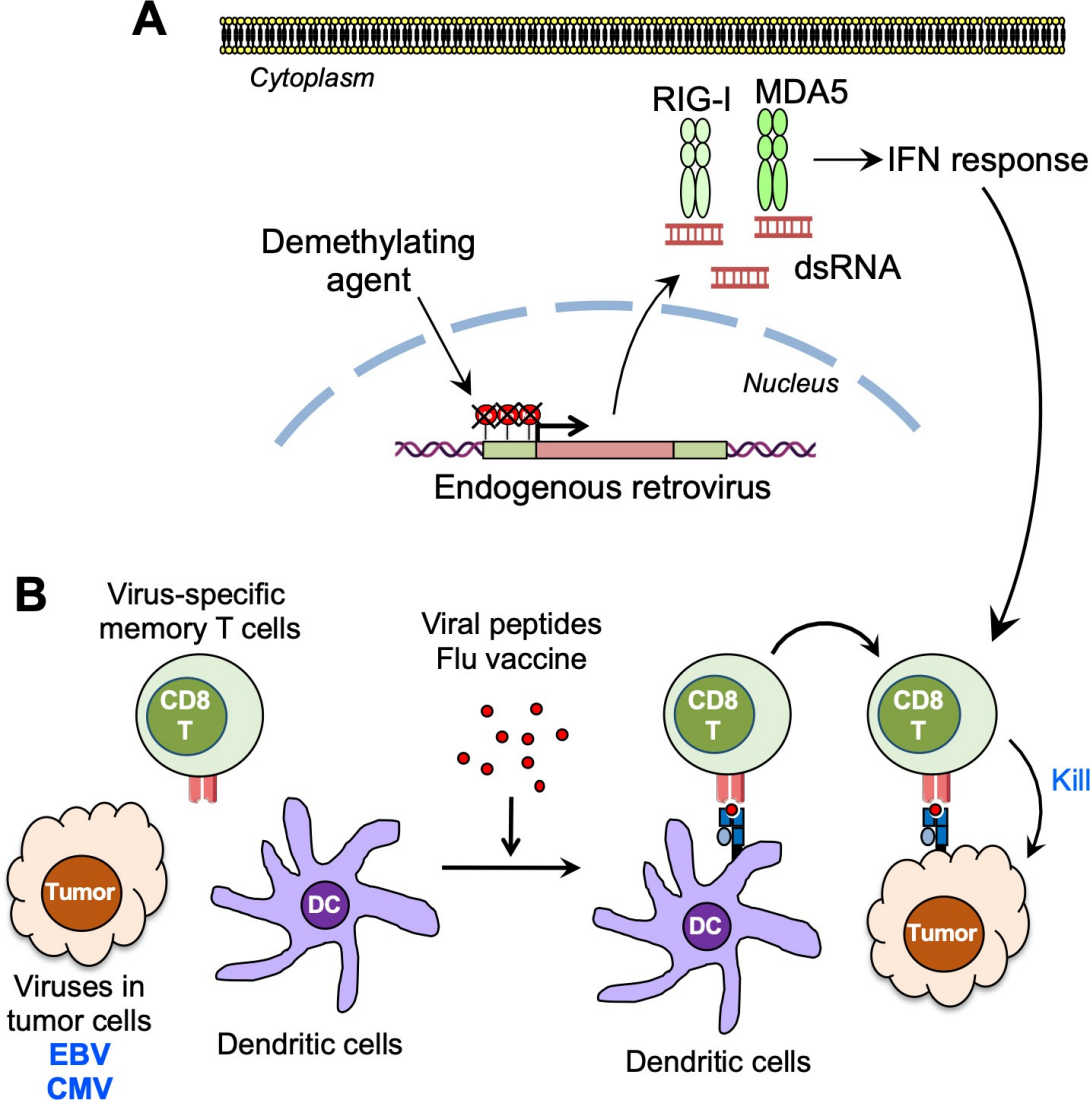
Antiviral immune responses sensitize antitumor immunotherapy. (A) Reactivation of ERVs by DNA demethylation induces an IFN response through cytosolic sensing of double stranded RNA. (B) Activation of virus-specific memory T cells induces antitumor CD8^+^ T cells through cross-presentation of viral peptides on DCs. CD8T, CD8^+^ T cells; CMV, cytomegalovirus; DC, dendritic cell; dsRNA, double stranded RNA; EBV, Epstein-Barr virus; ERV, endogenous retrovirus; Flu, influenza; IFN, interferon; MDA5, melanoma differentiation-associated protein 5; RIG-I, retinoic acid-inducible gene I.

Indeed, reactivation of endogenous retroviruses (ERVs) by DNMTi enhances susceptibility to anti-cytotoxic T-lymphocyte-associated protein 4 (CTLA-4) ICI therapy in the B16 mouse melanoma model, a model that has been shown to be highly resistant to ICIs (Fig 1A) [2]. DNMTi treatment significantly up-regulates the expression of intercellular adhesion molecule 1 (ICAM1), interleukin-1 receptor antagonist (IL-1RA), CXCL10, CCL2, and CCL5 and increases the percentage of effector CD8^+^ T and natural killer (NK) cells in the TME [25]. Interestingly, these antitumor effects induced by DNMTi completely disappear when the IFN-α/β receptor α chain (IFNAR1) is blocked, suggesting that type I IFN signaling is critical. The combination therapy of DNMTi and histone deacetylase inhibitors (HDACi) synergistically increases reexpression of silenced ERVs in cancer cells and increases sensitivity to the anti-programmed cell death 1 (PD-1) therapy in lung [26] and ovarian cancers [25]. Interestingly, however, the HDACi treatment alone has little effect, suggesting that DNA demethylation is necessary for ERV reactivation.

Consistent with these findings in mouse models, the signatures of antitumor effector function and anti-PD-1 responsiveness are also positively associated with HERV expression in human cancers [27]. In addition, Haffner and colleagues found that type I IFN signaling and the number of CD8^+^ T cells are significantly higher in seminomatous testicular germ cell tumor (TGCT) characterized by constitutive DNA hypermethylation when compared to several non-seminomatous TGCTs that have normal methylation levels [28]. Beyond ERV reactivation, DNMTi also induces antitumor immune responses by activating transcription of tumor antigens (e.g., cancer-testis antigens) and Th1-type chemokines (e.g., CXCL9 and CXCL10) [6, 29]. These results suggest that DNMTi could be used as a useful multi-tool for cancer immunotherapy.

### Cyclin-dependent kinase inhibitors

While it is well known that inhibitors of cyclin-dependent kinase (CDK) 4/6—the key regulators of the G1–S transition of the cell cycle—directly inhibit tumor growth by arresting cell cycle progression, recent studies have also discovered that CDK4/6 inhibitors can induce strong antitumor immune responses [30–32]. CDK4/6 inhibitor treatment reactivates ERVs, produces type III IFNs, suppresses the proliferation of regulatory T cells, and down-regulates the expression of the E2F transcription factor target genes including DNA methyltransferase 1 (DNMT1) [30]. Interestingly, CDK4 also directly interacts with and stabilizes DNMT1, thus further supporting the notion that CDK4/6 inhibitors would impair DNMT1 activity and reactivate ERVs [33].

In addition to CDK4/6 inhibition, inhibition of CDK9—a transcriptional activator recruited to promote RNA polymerase II—also induces ERV expression and antitumor immune activation without DNA demethylation [4]. A selective CDK9 inhibitor blocks phosphorylation of the chromosome remodeling protein SWI/SNF related, matrix associated, actin dependent regulator of chromatin, subfamily a, member 4 (SMARCA4), which epigenetically silences a set of genes including ERVs independently of DNA methylation [4]. Accordingly, ERV reactivation by treatment of CDK4/6 or CDK9 inhibitors significantly enhances the response rates of the current ICI therapies.

## How can quiescent antiviral immunity be reactivated to boost antitumor immunity?

The idea of using antiviral immunity for cancer treatment has been studied for over a century, and finally, the first oncolytic virus (Talimogene laherparepvec [T-VEC]) was approved by the Food and Drug Administration (FDA) in 2015 [34, 35]. Many different recombinant human viruses, including adenovirus, herpesvirus, poliovirus, reovirus, and poxvirus, are being used as oncolytic viruses in approximately 120 ongoing clinical trials to directly lyse tumor cells and indirectly stimulate antitumor immune responses (ClinicalTrials.gov) [36]. In addition to the oncolytic viruses, quiescent antiviral immune responses activated by viral peptides and vaccines can induce antitumor activity in the TME [5, 37].

Recent studies have shown that memory T cells specific to viruses in the human virome such as CMV and EBV are abundant in humans and can be activated by viral antigenic peptides [11, 37]. Although these host antiviral memory T-cell responses are not sufficient to eliminate life-long persistence of the viruses, treatment with viral peptides may be useful to awaken existing memory T-cell responses against exogenous viruses, such as CMV and EBV, persistent in the human virome to promote an antitumor response [37]. Accordingly, injection of CMV viral peptides significantly enhances the efficacy of the anti-PD-1 ligand 1 (PD-L1) therapy and suppresses tumor growth by activating an antiviral memory T-cell response (Fig 1B) [37].

The FDA-approved seasonal influenza vaccine greatly increases cross-presenting dendritic cells (DCs) and tumor antigen-specific CD8^+^ T cells in the TME (Fig 1B). These DCs and CD8^+^ T cells have been shown to play an important role in both antiviral and antitumor immune responses when the influenza vaccine is injected intratumorally [5]. Although adjuvants in vaccines normally boost immune responses against target pathogens, antitumor CD8^+^ T-cell responses are abrogated by adjuvant-enhanced regulatory B cells. These results suggest differential regulations of antiviral and antitumor immune responses despite their shared immune components and mechanisms. To further screen commercially available vaccines that activate the immunogenicity of cold tumors, Shekarian and colleagues tested 14 viral and bacterial vaccines and found that intratumoral injection of 2 rotavirus vaccines (Rotarix [GlaxoSmithKline Biologicals, Rixensart, Belgium] and RotaTeq [Merck, Kenilworth, NJ]) can activate antitumor immunity and aid in overcoming tumor resistance to ICI immunotherapies, suppressing tumor growth [38]. These results represent a potent mechanism to initiate key immune responses in tumors to assist in successful treatment.

Taken together, recent findings suggest that the awakening of antiviral host immune responses to the viruses in the human virome could be an effective force to fight against cancer.
